# Advances in Gene Therapy for Inherited Haemoglobinopathies

**DOI:** 10.3390/hematolrep18010004

**Published:** 2025-12-27

**Authors:** Anna B. Gaspar, H. Bobby Gaspar

**Affiliations:** 1 Kings College Hospital Foundation Trust, London SE5 9RS, UK; annagaspar5@gmail.com; 2 Molecular and Cellular Immunology, UCL Great Ormond Street Institute of Child Health, London WC1N 1EH, UK; 3 Orchard Therapeutics, London W6 8PW, UK

**Keywords:** inherited haemoglobinopathies, sickle cell disease, thalassaemia, gene therapy, gene editing

## Abstract

Haemoglobinopathies, including β-thalassaemia and sickle cell disease (SCD), are among the most common monogenic disorders worldwide and remain major causes of morbidity and early mortality. Historically, management of these life-altering diseases has relied on supportive treatment and symptom management and, although these treatments reduce symptoms and ease disease burden, they do not correct the underlying genetic defect. Allogenic haematopoietic stem cell transplantation (HSCT) has been the only established curative option; however, it comes with substantial risks that significantly restrict its applicability. Over the past two decades, haematopoietic stem cell (HSC) gene therapy for haemoglobinopathies has rapidly progressed from experimental proof-of-concept to approved therapies. Lentiviral gene addition approaches have demonstrated durable expression of functional β-like globin transgenes, achieving transfusion independence in β-thalassaemia patients and significant reductions in vaso-occlusive events in SCD patients. Alternative therapeutic approaches to promote HbF expression have proved to be highly successful. Gene silencing strategies targeting BCL11A have been successful clinically and, more recently, gene editing technologies such as CRISPR/Cas9 have enabled precise disruption of regulatory elements controlling γ-globin repression, leading to the approval of the first CRISPR-based therapy for SCD and β-thalassaemia. Emerging base editing technologies promise even more precise genetic modification and are advancing through clinical evaluation. Despite these advances, access to gene therapy remains restricted due to the need for highly specialised manufacturing, toxic myeloablative conditioning regimens, and high treatment costs. Ongoing improvements and adaptations in these areas are essential to ensure that gene therapies fulfil their potential as accessible, curative treatments for patients suffering from haemoglobinopathies worldwide.

## 1. Introduction

Haemoglobinopathies define a group of monogenic blood disorders characterised by the inheritance of abnormal haemoglobin (Hb) chains. They include thalassaemias and sickle cell disease (SCD) and seriously affect the health of up to 5% of the global population [1]. Together, they constitute the most common monogenic disorders worldwide.

Normal haemoglobin molecules are made up of polypeptide globin chains with iron-containing haem molecules, known as haemoglobin A (HbA). There are a number of different globin chains, each of which is coded for on separate genes. In healthy adults, HbA consists of two α-globin chains and two β-globin chains.

**Figure 1:**
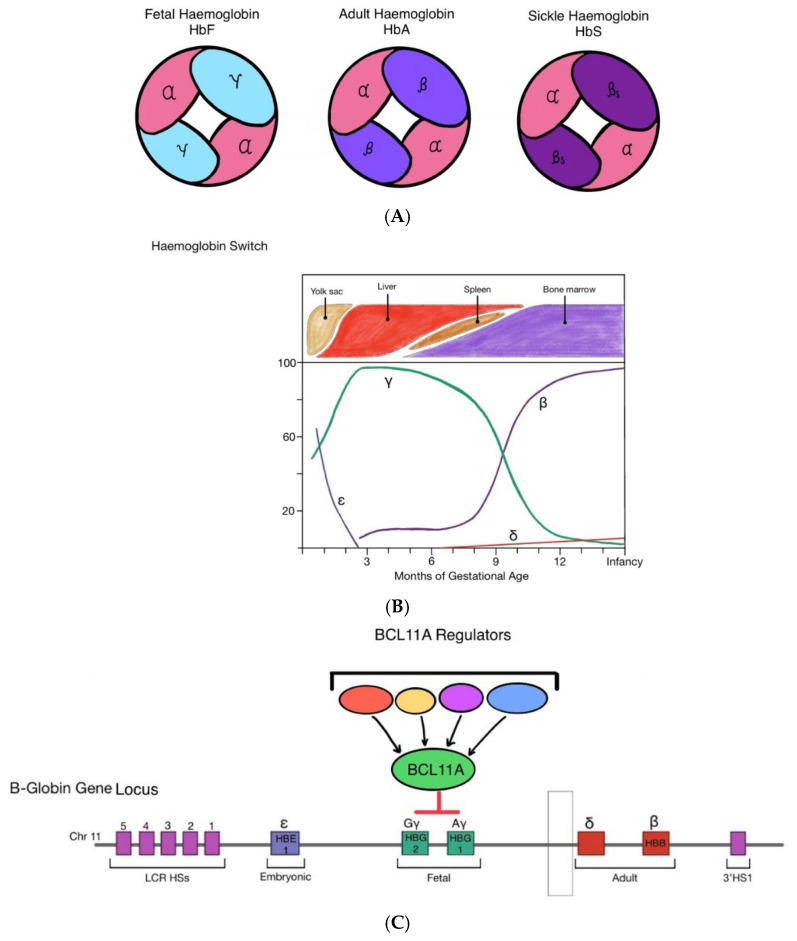
(**A**) Foetal haemoglobin (HBF) is made up of two α-globin chains and two γ-globin chains. In early infancy, adult haemoglobin (HbA) becomes the predominant form of haemoglobin, made up of two α-globin chains and two β-globin chains. In sickle cell disease, a mutation on the β-globin locus gives rise to pathogenic sickle haemoglobin (HbS) which aggregate and polymerise in red blood cells, making them stiff and deformed (sickled). (**B**) During foetal development in utero, γ-globin and thus HbF is most predominantly expressed to support foetal growth as it has greater affinity to oxygen than HbA. At approximately 32–36 weeks gestation, production of HbF decreases. Simultaneously, β-globin expression increases and HbA is produced in greater quantities, leading to a gradual transition from HbF to HbA shortly after birth. The onset of β-Haemoglobinopathies occurs postnatally, once the switch from HbF to HbA has taken place, as their mutations are confined to the β-globin gene locus. (**C**) BCL11A functions within a multiprotein transcriptional repression complex in erythroid precursors. Through this network of interacting proteins, BCL11A engages multiple regulatory sites across the β-globin locus. Although BCL11A does not directly bind the γ-globin genes, its activity is central to the developmental switch from foetal to adult haemoglobin. In adult-stage erythropoiesis, BCL11A expression drives the shift from γ-globin to β-globin production, thereby promoting the predominance of HbA over HbF.

However, in foetal and early neonatal life, haemoglobin F (HbF) is the predominant Hb molecule. Foetal haemoglobin is made up of two α-globin and two γ-globin chains (Figure 1A). The switch from HbF to adult haemoglobin (HbA) occurs within the first few months of life (Figure 1B).

Haemoglobin switching from HbF to HbA is regulated by the repression or activation of specific globin genes by transcription factors and the locus control region (LCR) on the 5′ end of the β-globin gene cluster [2]. BCL11A is a transcriptional repressor of γ-globin and plays a key role in facilitating the switch from HbF to HbA. Upon transcription, BCL11A silences γ-globin expression in erythroid cells through binding to specific promoter and enhancer regions on the γ-globin gene promoter (Figure 1C). The binding of BCL11A inhibits γ-globin expression, while other interactions promote the transcription of β-globin until β-globin levels surpass γ-globin. It is a crucial step in the transition from HbF expression to HbA [3].

Hereditary persistence of foetal haemoglobin (HPFH) is a benign haematological condition characterised by the persistent expression of foetal γ-globin into adulthood. This results from either large deletions downstream of the γ-globin gene within the β-globin cluster (deletional HPFH mutations) or mutations within the γ-globin gene promoters (non-deletional HPFH mutations). The inheritance of both HPFH and SCD or thalassemia mitigates disease severity, as persistent γ-globin production compensates for the deficient or structurally abnormal β-globin chains [4]. Infants with HPFH alongside SCD have fewer symptoms of disease, and their erythrocytes take more time to sickle and do not sickle as profoundly [5]. As suggested by the therapeutic effects of hydroxyurea treatment, and the protective nature of persistent foetal haemoglobin, inducing the expression of foetal haemoglobin (HbF) postnatally through gene therapy has been postulated to be an effective method of treating inherited haemoglobinopathies.

There are a number of different forms of thalassaemia but the most severe is β-thalassaemia, which occurs when a mutation arises in the β-globin gene that leads to reduced, or total lack of production of, HbA. Patients with the most severe form of β-thalassaemia may suffer from completely ineffective erythropoiesis and subsequently chronic haemolysis. Patients are often blood transfusion-dependent. also termed transfusion-dependent thalassaemia (TDT), requiring regular iron chelation therapy to reduce the risk of iron overload.

SCD arises from a single base pair point mutation in the β-globin gene known as haemoglobin S (HbS). When exposed to a deoxygenated environment, this amino acid substitution causes pathogenic globin chains to undergo polymerisation and become misshapen or ‘sickled’ [6]. Patients with SCD suffer acute vaso-occlusive crises, chronic haemolytic anaemia, progressive multi-system organ failure, and have an overall reduced life expectancy of 45–50 years [7].

The first pharmaceutical approach for the treatment for haemoglobinopathies was via the life-long administration of hydroxyurea, which switches production of defective β-globin to γ-globin. In more recent years and for the most severe patients, allogenic bone marrow transplantation, which allows effective erythropoiesis from the wild-type engrafted allogenic haematopoietic stem cells (HSCs), has been the only curative treatment option for these diseases. This requires transplantation from either a human leukocyte antigen (HLA)-matched sibling donor, matched unrelated donor, umbilical cord blood transplantation, or haploidentical transplantation. However, depending on the donor source, these options carry a significant risk of graft rejection, graft failure, and graft versus host disease (GVHD), which can be fatal.

Improved understanding of the molecular mechanisms underlying the development and transition from HbF to HbA and the specific mutations that give rise to haemoglobinopathies has broadened the horizons for a safer and potentially curative treatment for these diseases. The trial and development of autologous HSC gene and gene editing therapies (HSC-GT), which aim to insert a working copy of the faulty gene or modify specific genomic sequences, have potential to be a safe and curative treatment for patients for these monogenic diseases. The use of autologous HSCs clearly avoids the risk associated with using allogeneic donors, but the procedures still require the use of myeloablative conditioning regimens.

Developing an effective genetic therapy for the treatment of haemoglobinopathies has proved to have many challenges, including the complex regulation of globin gene expression. β-globin expression is dependent on erythroid-specific transcription controlled by the interaction between β-globin LCRs with β-globin promoter sequences, thus enabling β-globin transcription and triggering the switch from foetal to adult haemoglobin expression shortly after birth [8]. However, given the unmet need, the understanding of the molecular pathophysiology of disease, advances in genetic modification techniques and learnings from advances in gene therapy for other monogenic haematological diseases, there are now a number of gene therapy options available for haemoglobinopathies. These include gene addition, gene silencing and gene editing strategies, using a variety of molecular tools (summarised in Table 1).

## 2. Gene Addition Approaches

The first gene therapies for haemoglobinopathies were developed in the early 2000s using lentiviral vectors (LVs), which were known for their ability to transduce haematopoietic HSCs efficiently and, over many years, have shown a high safety profile when the appropriate promoter constructs are used. These were ‘gene addition’ strategies in which LVs were designed to deliver a functional β-globin gene, alongside a specifically designed promoter sequence to allow the appropriate, near physiological, expression of β-globin in erythrocyte development.

Another important innovation was the use of a modified β-globin transgene designed to treat both TDT and SCD. γ-globin has naturally superior anti-sickling properties when compared to β-globin, despite the very few differences in amino acid residues between γ-globin and β-globin chains [20]. The superior inhibitory effect of γ-globin on HbS polymerisation is thought to be due to a key difference in a critical residue at position 87, where the change from Threonine (T) to Glutamine (Q) reduces the ability of HbS to form rigid polymerising fibres [21]. Gene addition therapies were first trialled using an LV (denoted HPV569), which encoded a β-globin transgene with a specific T87Q mutation possessing anti-sickling properties, together with regulatory elements incorporating mini LCR elements designed to enhance erythroid expression and prevent unwanted transactivation of adjacent genes. It became the first clinical trial to be initiated for gene therapy to treat both transfusion-dependent β-thalassemia (TDT) and SCD [22]. In the first patient treated in this study, transfusion independence was achieved in part due to the expansion of a benign dominant clone, in which the integrated vector caused transcriptional activation of growth factor HMGA2 in erythroid cells. The expansion remained benign and was not seen again in subsequent patients.

To improve transduction efficiency and the vector copy number (VCN) in transduced HSCs, improvements to this initial vector were made, including replacing the wild-type HIV-1 U3 region with a strong constitutive CMV promoter to drive higher expression levels of packageable RNA in producer cells and the removal of redundant insulator sequences. The BB305 vector was then tested in further clinical trials for SCD and TDT; initial studies showed that two years after the infusion of gene-modified HSCs, all 22 TDT patients recruited to the trials were either transfusion independent or had a significantly reduced need for long-term transfusions [23]. In 2022, the FDA announced the approval of Zynteglo for the treatment of TDT. With 4 years of follow-up data available, the genetic medicine, which induces the expression of β-globin transgene T87Q, achieved transfusion independence in 89% of TDT patients treated [9].

SCD patients did not display such significant clinical improvement in these initial trials due to the low yield of CD34+ HSCs and low VCN. Further trials were carried out using the BB305 LV to treat SCD with optimised conditioning, which involved pre-harvest blood transfusions, improved HSC mobilising regimens prior to apheresis, higher doses of myoablative busulphan, and improved manufacturing regimens. Patients enrolled in this trial (HBG-206) exhibited β-globin transgene T87Q expression that was equal, or superior to, HbS expression as early as 3 months after infusion [24]. However, while no patients suffered from graft rejection or failure, some patients experienced serious adverse effects (AEs) related to the HSC harvesting and conditioning regimens, suggesting further modifications needed to be made to optimise the safety profile of this treatment [25]. In 2020, a phase 3 HGB-210 trial (NCT04293185) was initiated to investigate the efficacy and safety of LentiGlobin BB305 in paediatric patients aged 12–18 years. In both the HGB-206 and HGB-210 trials, patients received a single infusion of autologous CD34+ HSCs that had been transduced ex vivo with the lentiviral vector encoding the βA-T87Q globin gene. This approach facilitated stable production of HbAT87Q and was associated with a marked reduction in the frequency of vaso-occlusive events (VOEs) in 88.2% of patients as little as 18 months following treatment [10]. These trials led to the FDA approval of Lyfgenia in 2023 for the treatment of SCD.

An alternative approach was tested in an alternative study using the GLOBE lentiviral vector to express a native β-globin in autologous CD34+ HSCs. The native transgene in this vector was able to address TDT but was not designed for the treatment of SCD. Patients were treated via a different myoablative regimen using treosulfan and thiotepa. Six out of nine patients achieved transfusion independence, and the remaining patients had decreased transfusion reliance [26]. Further development of this approach is awaited.

Gene addition of γ-globin using lentiviral vectors has also shown promising anti-sickling effects for the treatment of haemglobinopathies. The sGbG LV is a vector designed to carry a β-γ-globin hybrid gene (I8Hβ/γW) encoding a chimeric γ-globin gene replaced with intron 2 and splicing signals from β-globin [27]. This successfully showed high expression of γ-globin mRNA in adult globin-expressing erythrocytes. The application of this vector in humanised mouse models showed amelioration of disease activity with HbF expression as little as 10% [28]. The same vector demonstrated HbF expression of up to 15% in non-human primates [29]. This vector was further modified by altering the 16th codon in γ-globin exon 1 to change glycine (G) to aspartic acid (D), generating the GbGM lentiviral vector gene that expresses γG16D-globin, which has greater anti-sickling activity. The vector has been approved for phase 1 and 2 trials of gene addition in SCD patient CD34+ cells with a reduced-intensity conditioning regimen using melphalan, as opposed to a Busulfan-based regimen. Despite lower VCNs, there was a high reduction in VOEs in the seven patients treated, suggesting that the more potent anti-sickling gene may have contributed to therapeutic efficacy [11].

## 3. Gene Silencing

BCL11A is a transcriptional repressor of γ-globin, which plays a key role in regulating the transition from HbF expression to HbA. In 2020, a lentiviral vector was developed encoding a short hairpin RNA (shRNA), BCH-BB694 to knock-down BCL11A through RNA interference, thus enabling the reactivation of the γ-globin gene and, therefore, HbF production, and reducing the expression of the sickling pathogenic β-globin [30]. BCH-BB694 is being tested in a clinical trial for the treatment of SCD, and preliminary data from six patients show HbF production in up to 94% of red blood cells and absent or significantly reduced disease burden in all six patients [12].

## 4. Gene Editing

Gene editing involves techniques designed to alter the DNA sequences at specific genomic loci. Editing can be affected by molecular tools that can create targeted double-stranded breaks (DSBs), thereby triggering endogenous repair systems that can either deactivate a desired gene or, by the provision of a DNA template, allow for restoration of the wild type or desired sequence. More recent base editing technologies allow for even more precise chemically induced substitution of one base for another at specific sites, without the need for double-stranded breaks. When compared to gene addition therapies, gene editing tools may have an advantage, as they avoid the risk of random gene integration, and through either repair of mutation in situ or insertion of exogenous gene sequences within the targeted gene loci, the modified gene remains under the control of endogenous regulatory systems.

CRISPR (clustered regularly interspaced short palindromic repeats)/Cas9 (CRISPR-associated protein 9) is one of the preferred gene editing tools due to its ease of use, high specificity, and prominent activity levels across a range of cell types. The CRISPR-Cas9 system uses single-guide RNAs (sgRNAs) to direct Cas nucleases to target specific DNA sequences and create DNA strand breaks, thereby triggering repairs, insertions, or deletions in precise genomic regions [31]. The DSBs are repaired via nonhomologous end-joining (NHEJ) or homology-directed repair (HDR). In NHEJ, frameshift mutations, either insertions or deletions (indels), are generated during repair of homologous and nonhomologous DNA strands created by Cas9. However, this process is susceptible to error due to the unpredictable nature of indels and can be used as a deliberate mechanism to ‘knockout’ gene expression and function. Alternatively, HDR uses homologous exogenous DNA templates to repair the DSBs, allowing for controlled deletions, substitutions or insertions via the introduction of a pre-selected sequence and is a mechanism for repairing a mutated gene to a wild-type or desired sequence [32].

CRISPR-Cas9 technology has been used to target and prevent the expression of BCL11A through edits at the erythroid-specific enhancer region, thereby allowing γ-globin expression and increasing HbF synthesis. In 2021, by complexing the CRISPR/Cas editing machinery with a lipid nanoparticle delivery system, ex vivo modification of CD34+ cells from healthy donors achieved the desired genetic disruption of BCL11A in 80% of cells with no detectable off-target editing. In a subsequent clinical trial, two patients (one SCD, one TDT) received autologous CD34+ HSCs, edited with CRISPR-Cas9 after myeoloabative conditioning, resulting in high levels of allelic disruption and HbF expression. Both patients achieved transfusion independence, and the SCD patient was free of vaso-occulsive events [33]. These promising clinical results paved the way for the CLIMB THAL-111 and CLIMB SCD-121 trials, testing the efficacy of CRISPR–Cas9 gene editing of autologous CD34+ (Exa-cel) to treat TDT and SCD, respectively. Further, 52 TDT patients were enrolled to phase III of the CLIMB THAL-111 trial and received busulfan conditioning prior to treatment. Transfusion independence was achieved in 91% of patients as well as pan-cellular (94%) expression of HbF. The safety profile of the treatment was consistent with that of busulfan conditioning and autologous transplant, and there was no evidence of oncogenesis or clonal proliferation [34]. Additionally, 44 SCD patients were treated as part of CLIMB SCD-121 and, at the time of follow-up, none of the 30 patients with available interim analysis data required hospitalisation for vaso-occlusive events, and 97% were completely VOE-free [13]. Then, 6 months post-treatment, the percentage of red cells expressing HbF was >93%, and all patients achieved transfusion independence. As with CLIMB THAL-111, the safety of treatment was consistent with that of conditioning and transplantation [13]. In 2023, exa-cel (trade name Casgevy) was approved by the FDA and MHRA for the treatment of both SCD and β-thalassaemia, making it the first approved CRISPR-Cas9-based gene therapy.

CRISPR-Cas9 technology has also been adapted to replicate the HPFH mutation in patients with SCD and thalassaemia. There are various genotypes of HPFH; however, they all involve a mutation or deletion of the sequences between the γ and δ genes on the β-globin locus. These sequences encode important binding sites of BCL11A that facilitate the repression of γ-globin expression [35]. In 2016, an RNA-guided Staphylococcus aureus Cas9 nuclease (SaCas9) was developed to excise a 13-kb segment of the γδ-intergenic sequence in CD34+ HSCs to mimic the inherited HPFH deletion with very good efficacy and no off-target gene editing. Results suggest that these edited HSCs express γ-globin at a level comparable to that seen in naturally occurring HPFH [36]. This paved the way for a clinical trial evaluating OTQ923, a CRISPR-Cas9 product with targeted disruption of HBG1 and HBG2 promoters on the β-globin gene cluster where BCL11A binds. The trial saw three SCD patients treated with OTQ923. HbF expression and subsequent improvement in disease activity were seen in all three patients; however, several adverse events occurred relating to myeloablative conditioning and the underlying disease [14].

Other gene editing approaches include the use of zinc-finger nucleases (ZFNs). ZFNs are chimeric nucleases consisting of a DNA binding domain and a DNA cleavage domain. Together, they enable site-specific DNA mutagenesis via indels or base substitution by creating a DSB at a preprogrammed target site with high efficiency and minimal off-target editing [37]. ZFNs have been developed to target the GATAA-binding motif in the BCL11A erythroid-specific enhancer to reactivate HbF in a gene therapy known as BIVV003. Preliminary results from clinical trials of BIVV003 to treat SCD have shown promising results. Five of six patients evaluated at interim analysis had an overall increase in HbF production, and no severe VOCs have been observed [15].

## 5. Base Editing

Base editing is a more recent genetic technology, which uses base editors (a fusion of a deactivated Cas9 protein and a deaminase enzyme) and sg-RNAs to make single-nucleotide changes in target DNA sequences. It offers advantages over CRISPR-Cas9 gene editing strategies as it does not rely on the creation of double-stranded DNA breaks and subsequent non-homology directed repair, therefore reducing the risk of introducing errors through indels or off target effects. Base editing approaches to treat SCD aim to increase the expression of HbF by editing transcriptional repressor BCL11A, or BCL11A binding regions HBG1 and HBG2, as well as editing binding sites of transcriptional activators of γ-globin genes [38].

The BEACON phase I/II trials tested the use of BEAM-101, a base editing product composed of CD34+ HSCs edited to introduce A-G substitutions into HBG1 and HBG2 to prevent BCL11A binding, thus promoting HbF expression [16]. The trial supported the safety of BEAM-101 treatment, in line with that of busulfan-conditioning regimens and autologous HSC transplantation. Preliminary data suggest patients who received BEAM-101 demonstrated a significant and sustained increase in HbF expression (>60%) as well as a reduction in HbS percentage (<40%). No patients enrolled in the trial experienced any vaso-occlusive events following treatment; however, longer-term follow-up from this trial is required.

In an alternative approach, BEAM-102 was developed to convert the pathological sickling allele to Hb G-Makassar, a benign, naturally occurring, non-sickling β-globin variant. In pre-clinical trials, this engineered adenine base editor achieved conversion of >70% sickle alleles to Makassar alleles in CD34+ β^s^/β^s^ cells in vitro [17].

A novel base editing approach was designed to revert the most common β-thalassemia mutation, IVS1-110 (G > A), back to the wild-type sequence using the SpRY-ABE8e adenine base editor. In pre-clinical models, delivery of this system via RNA proved to be safe, achieving approximately 80% correction of the defective gene in HSCs from β-thalassemia patients. This technique successfully restored β-globin synthesis in erythroid cells derived from the edited progenitors, resolving the lack of erythropoiesis that is characteristic of β-thalassemia [18].

A different base editing strategy for the treatment of β-thalassemia targeted the IVS1-110 splice-site mutation using adenine base editors (ABEs). This technique achieved up to 90% editing of the upstream regulatory component crucial for the pathogenic splicing process, achieving correction in patient-derived CD34+ cells [19].

Other pre-clinical studies have investigated a transformer base editor (tBE)—a cytosine base editor characterised by superior precision and minimal off-target activity—to modify promoter regions of HBG1 and HBG2. Targeted disruption of six regulatory motifs within these loci resulted in a substantial elevation of γ-globin expression, surpassing the levels achieved through Cas9-mediated editing or conventional base editing platforms [39]. Importantly, the genetic modifications introduced by tBE were maintained in HSCs, suggesting long-term therapeutic potential and a promising therapeutic approach for the treatment of haemoglobinopathies; however, these models are yet to be tested in vivo.

## 6. Considerations and Discussion

A number of different genetic modification approaches have now been able to demonstrate a long-term, if not permanent, correction of the TDT and SCD clinical phenotypes and allow patients and physicians alike to envisage a future whereby all patients could be offered a curative option. However, while clinical effectiveness and regulatory approval have been demonstrated, the reality of treating all patients has proved to be very different and, to date, the uptake of approved therapies has been limited and fallen well short of initial expectations. The hurdles to more universal adoption of these transformative therapies are multifactorial but can be broken down into issues associated with the process and the reimbursement and access environment.

HSC gene therapy remains, at present, an ex vivo procedure where autologous cells have to be harvested from the patient before being shipped to a manufacturing site, genetically modified, cryopreserved and then shipped back to the transplant site before being reinfused after the patient receives a conditioning regimen. This process requires patients going to qualified treatment centres where there is expertise in transplant procedures and, therefore, requires patients travelling, sometimes together with their families, from their own centre to the qualified treatment centre. In addition, following conditioning, there is a period of haematopoietic recovery, which can take anywhere between 3 and 6 weeks, during which patients are hospitalised. For adult patients with families and careers, this is a significant undertaking, and given that some patients have been maintained on regular transfusions and maintenance therapies, the absolute need to undergo the gene therapy procedure with its inherent short-term risks and time and travel obligations has to be balanced against the current standard of care, which has long-term risk but may be seen to be acceptable in the short to medium term.

Specific procedural issues are largely associated with the mobilisation of cells for harvest and the conditioning procedure. The majority of HSC gene therapies have moved from the collection of HSCs directly from the bone marrow to collection from peripheral blood via leukapheresis. For the latter, HSCs have to be mobilised from the bone marrow to the peripheral blood using mobilising agents, the most popular of which have been G-CSF (granulocyte colony stimulating factor) and Plerixafor. For SCD patients, the use of G-CSF has been associated with severe vaso-occlusive crises, multiorgan failure, and even death. For these reasons, G-CSF is contraindicated as a mobilising agent for SCD HSC gene therapy unless deemed absolutely necessary [40]. The use of Plerixafor alone is now the standard regimen for SCD patients and, in the vast majority of cases, provides enough CD34+ cells for a successful gene-modified cell graft.

The need for an intensive conditioning regimen to deplete resident mutated HSCs in the bone marrow, thereby ‘making space’ for incoming gene-modified cells, has been associated with toxicities that are difficult to overcome. In addition to the hospitalisation described above, there are toxicities such as mucositis, veno-occlusive disease and infertility, which are related to the most commonly used conditioning agent, Busulfan. Other chemotherapy agents, such as Treosulphan (in combination with Thiotepa) and Melphalan, have been used in HSC GT protocols for haemoglobinopathies [11,26], but each come with their own side effect profile. In the case of Melphalan, the dose used in the phase I/II trial [11] was designed to limit toxicity but came at the expense of decreased myeloablation and subsequent reduced engraftment of modified cells and, therefore, a lower VCN in the periphery following gene therapy.

More specifically, in trials of HSC GT for SCD using chemotherapy conditioning, two cases of myelodysplasia and acute myeloid leukaemia were seen. The first participant developed MDS 3 years after treatment, followed by transformation to AML with monosomy 7; the second participant developed AML 5.5 years after therapy. In the first case, no vector insertion was seen in AML blasts and, in the second case, although vector insertions were seen in the clonally expanded cells, the insertional sites were not deemed to be pathogenic. Genetic testing identified other genomic changes that could drive leukaemogenesis, including RUNX1, PTPN11 mutations, monosomy 7 and partial loss of 11p [41]. The presence of somatic mutations was probably multifactorial but may be related to the extensive proliferation of HSCs that follows chemotherapy conditioning, generating proliferative stress that may lead to the rise and accumulation of mutations as part of the normal engraftment process.

The need, therefore, to move from the current intensive chemotherapy-based conditioning regimens to less intensive protocols is the subject of ongoing study to make HSC GT more acceptable not only for SCD patients but also for other diseases currently targeted by HSC GT. Areas of investigation are numerous but most advanced are the use of monoclonal antibodies, which specifically target HSCs and will allow depletion specifically of these cells without associated toxicities on the lung, liver, GI tract, reproductive and other organs. A favoured and advanced approach is the use of anti-CD117 (or anti-c-kit) antibodies that target a receptor on the surface of HSCs and progenitors. Murine studies initially demonstrated the effective depletion of HSCs and successful haematopoietic reconstitution following CD117-targeted conditioning with limited toxicities [42]. Subsequent advancement to human clinical trials has demonstrated that anti-CD117 targeting alone is insufficient for full HSC depletion and subsequent multi-lineage reconstitution [43]. Approaches combining the use of anti-CD117 with other antibodies are in pre-clinical development [44].

Standard patients’ access to approved medicines is dependent on financial agreements between manufacturers and reimbursement agencies, which can be insurance providers or national agencies, depending on location. The process typically involves a health technology assessment to confirm the clinical and cost-effectiveness of a medicine followed by negotiation over the price. The one-time administration and long-term clinical benefit of HSC gene therapies, together with high development costs and complex manufacturing, have led to gene therapies being priced in the millions of dollars. For some rare disease indications, where there are limited or no treatment options, reimbursement agencies have accepted these prices. However, where there are alternative effective treatment options available, there have been significant differences between the manufacturers’ price and the price reimbursement agencies are willing to pay. Bluebird bio withdrew Zynteglo from the European market due to difficulties in reaching a commercial agreement on its high price with European governments, despite its conditional EU approval in 2019. The company cited the inability of European authorities to recognize the value of the gene therapy at the price it sought, which led it to focus its resources on the U.S. market, where it found a more favourable reimbursement path. More recently, agreements have been reached for Casgevy in both US and EU markets.

One major issue that remains to be addressed is access to these gene therapies for the vast majority of patients worldwide living with TDT and SCD. For SCD, millions of individuals live with the disease in sub-Saharan Africa [45], yet there is virtually no access in these regions for gene therapies. Given the complex manufacturing, the procedural aspects associated with delivering ex vivo therapies, and the high costs, there are major hurdles to overcome before such transformative therapies can reach the majority of patients in need.

Ultimately, and hopefully, many of these hurdles may be overcome through technological innovations. There is a significant body of work looking at the ability to deliver gene editing technology in vivo. CRISPR/Cas and base editing machinery can already be delivered via lipid nanoparticles to hepatocytes to delete specific genes (e.g., KLKB1 for hereditary angioedema and PCSK9 for familial hypercholesterolaemia), with promising results in clinical trials [46,47]. The ability to deliver machinery into HSCs in vivo is more difficult and would require the initial mobilisation of HSCs into the periphery and the infusion of LNPs that could specifically target HSCs and deliver editing machinery. Pre-clinical work in this area is advancing [48], and much still needs to be overcome to make this efficient and safe. However, such an approach may overcome the issues associated with the need for conditioning, ex vivo manufacturing complexities and its associated costs and potentially deliver the promise of these therapies to a wider geographical population.

## Figures and Tables

**Table 1:** Summary of gene therapy approaches for monogenic haemoglobinopathies.

Treatment	Mechanism of Action	Status
Gene addition	Lentiviral addition of T87Q [9,10]	Approved gene therapy
	Lentiviral addition of γ-globin [11]	Clinical trial
Gene Silencing	Lentiviral knockdown of BCL11A [12]	Clinical trial
Gene Editing	CRISPR/Cas9 editing of BCL11A erythroid enhancer [13]	Approved gene therapy
	CRISPR/Cas9 editing of HBG1/2 [14]	Clinical trial—discontinued
	ZFN editing of GATAA [15]	Clinical trial
Base Editing	Base editing of HBG1/2 [16]	Clinical trial
	Conversion to Hb-Makassar [17]	Pre-clinical trial
	Correction of IVS1-110 mutation back to wildtype [18,19]	Pre-clinical trial

## Data Availability

The data in this review are available in the referenced original manuscripts.
